# Retrospective data on causes of childhood vision impairment in Eritrea

**DOI:** 10.1186/s12886-017-0609-x

**Published:** 2017-11-22

**Authors:** Rajendra Gyawali, Bharat Kumar Bhayal, Rabindra Adhikary, Arjun Shrestha, Rabindra Prasad Sah

**Affiliations:** 1 Department of Optometry, Asmara College of Health Sciences, Asmara, Eritrea; 2 Reyukai Eiko Masanuga Eye Hospital, Banepa, Nepal; 3 Children’s Hospital for Eye, ENT and Rehabilitation Services, Bhaktapur, Nepal; 4 Berhan Aiyni National Referral Eye Hospital, Asmara, Eritrea

**Keywords:** Childhood blindness, Vision impairment, Avoidable blindness, Eritrea

## Abstract

**Background:**

Proper information on causes of childhood vision loss is essential in developing appropriate strategies and programs to address such causes. This study aimed at identifying the causes of vision loss in children attending the national referral eye hospital with the only pediatric ophthalmology service in Eritrea.

**Methods:**

A retrospective data review was conducted for all the children (< 16 years of age) who attended Berhan Aiyni National Referral Eye Hospital in five years period from January 2011 to December 2015. Causes of vision loss for children with vision impairment (recorded visual acuity less than 6/18 for distance in the better eye) was classified by the anatomical site affected and by underlying etiology based on the timing of the insult and causal factor.

**Results:**

The medical record cards of 22,509 children were reviewed, of whom 249 (1.1%) were visually impaired. The mean age of the participants was 7.82 ± 5.43 years (range: one month to 16 years) and male to female ratio was 1:0.65. The leading causes of vision loss were cataract (19.7%), corneal scars (15.7%), refractive error and amblyopia (12.1%), optic atrophy (6.4%), phthisis bulbi (6.4%), aphakia (5.6%) and glaucoma (5.2%). Childhood factors including trauma were the leading causes identified (34.5%) whereas other causes included hereditary factors (4%), intrauterine factors (2.0%) and perinatal factors (4.4%). In 55.0% of the children, the underlying etiology could not be attributed. Over two-thirds (69.9%) of vision loss was potentially avoidable in nature.

**Conclusion:**

This study explored the causes of vision loss in Eritrean children using hospital based data. Cataract corneal opacities, refractive error and amblyopia, globe damage due to trauma, infection and nutritional deficiency, retinal disorders, and other congenital abnormalities were the leading causes of childhood vision impairment in children attending the tertiary eye hospital in Eritrea. As majority of the causes of vision loss was due to avoidable causes, we recommended primary level public health strategies to prevent ocular injuries, vitamin A deficiency, perinatal infections and retinopathy of prematurity as well as specialist pediatric eye care facilities for cataract, refractive errors, glaucoma and rehabilitative services to address childhood vision loss in Eritrea.

## Background

Childhood vision loss is a priority area of disease control targeted by Vision 2020: the right to sight global initiative for elimination of avoidable blindness [1]. This priority is not only because the children with blindness have a lifetime of disability ahead but also because they suffer from substantial psychological, educational and economic impact [2]. The estimates of the number of children with blindness in 2010 suggests that there are 1.26 million children worldwide who are blind, 0.42 million of whom live in sub-Saharan Africa [3]. While the estimated number of blind children has declined or at least stabilized in other parts of the developing world, in sub-Saharan Africa, this number has increased by 31% over the past 10 years [3]. Furthermore, many of the causes of blindness in children are either preventable or treatable, and are also related to child mortality [4].

There is a wide global and regional variation in causes of blindness in children based on the country’s socioeconomic status [4]. While corneal scarring due to infection and vitamin A deficiency (VAD) are the leading causes in low-income countries, the lesions of the central nervous system are the predominant causes in high-income countries. In all regions of the world, cataract, retinal diseases, and congenital abnormalities affecting the whole globe are important causes of blindness [4]. Proper information on locally predominant causes of vision loss in children is important as it will allow authorities to develop appropriate strategies to address such causes by allocating available limited resources to appropriate preventive and curative services [5]. However, population-based study to gather such information requires large sample size and are therefore expensive, time and resource intensive. Thus, the most of the available data on the causes of blindness in children from developing countries have been obtained from examining children in schools for the blind, although this method is also potentially biased because very few of the blind children attend such schools [4]. Other methods to collect data on childhood blindness include key informant methods [6], blindness registry and hospital based studies [7–9]. Hospital records of the children can provide the information on childhood vision loss with minimum cost, human resource and time spent. Such information can be the first information or complement the existing information required as evidence to design appropriate blindness control programs in resource limited countries like Eritrea.

Until recently, there was no information available on causes of childhood vision loss in Eritrea. In our attempt to understand the causes of vision impairment in Eritrean children, we conducted a survey of children attending the sole school for the blind in Eritrea [10, 11] and a retrospective study of visually impaired children at the national referral eye hospital. These studies together provide much needed information on childhood blindness in Eritrea and are believed to assist authorities in the development of appropriate strategies and programs. This report presents the findings of the analysis of hospital based data on childhood blindness in the country.

## Methods

Ethical approval was obtained from Health Research Proposal Review and Ethical Clearance Committee at Ministry of Health for a retrospective study design. Medical Record Cards (MRCs) of 22,509 children (aged 16 years or less) attending Berhan Eye Hospital (BEH) between January 2011 and December 2015 were analyzed. The records of medical history, family history and ophthalmological evaluation were reviewed. BEH is the national referral eye hospital in Eritrea which hosts the country’s only pediatric eye care facilities. Patients attending outpatient department at BEH undergo a complete ocular and vision examination including external examination with either torch light and magnifying loop or slit lamp, fundoscopy, refraction, and binocular single vision assessment, where cooperation is adequate. Visual acuity (VA) is assessed using Snellen chart and pictorial charts whereas the fixation pattern (central, steady, maintained) is taken into account in patients whose visual acuity cannot be measured.

The study population included the children with visual acuity in the category of vision impairment (presenting VA less than 6/18 in better eye). If VA was not recorded in a MRC, signs of poor vision, like searching nystagmus, ocular bobbing or oculo-digital massage were considered. For the analysis purpose, vision loss was categorized based on the 10th edition of International Classification of Disease [12]: no vision loss: VA ≥ 6/18, visual impairment: VA < 6/18 but ≥ 6/60, severe visual impairment: VA < 6/60 but ≥ 3/60, blindness: VA < 3/60. Further categories of “believed sighted” and “believed blind” were added in this classification to include children where VA was not measured in Snellen’s fraction. Incomplete MRCs where both presenting VA and a diagnosis were not mentioned were excluded from analysis.

Based on the details mentioned on the MRCs, the causes responsible for vision loss for each eye and each child as a whole were classified on both anatomic and etiological basis according to the guidelines provided along with the WHO/PBL record for the examination of children with blindness and low vision [13]. The anatomical categories included whole globe (including glaucoma, microphthalmos, anophthalmos and phthisis bulbi), cornea, lens, uvea, retina, optic nerve and normal globe (including refractive error/amblyopia, nystagmus and cortical blindness), and allowed the examiner classify the location or the part of the eye affected. In cases with multiple abnormalities, the condition which likely contributed most to the vision loss, was preventable in nature and had the most recent occurrence was selected as the principal site. The etiologic classification was primarily based on the stage in the child’s development when the injury leading to visual loss happened. The etiologic categories included: hereditary diseases, intrauterine factors, perinatal/neonatal factors, postnatal/infancy/childhood factors and unclassifiable factors (time of injury and causal factors unknown). Because in many cases, the family history and other details were not available on the MRCs and diagnostic laboratory tests were not performed, many of the factors designated were marked ‘suspected’ rather than ‘definite’. For the analysis purpose, the causes responsible for the vision loss in the child as a whole were considered.

The data were managed and analyzed using SPSS version 19 software (IBM Corporation, Chicago, IL). Multinomial logistic regression analysis was used to explore the difference in causes of vision loss among demographic groups. A *p*-value <0.05 was considered statistically significant.

## Results

Records of 249 children with confirmed or suspected vision impairment were identified and further analyzed (1.1% of 22,509 total children seen in the study period). The demographic details identified in records are presented in Table 1. The mean age at presentation was 7.82 ± 5.43 years (range: one month to 16 years) and male to female ratio was 1:0.65.

**Table 1:** Baseline characteristics of the children (*N* = 249)

Baseline characteristics		Number	Percentage
Age (years)	<5	84	33.7
	5–9	62	24.9
	≥10	103	41.4
Sex	Male	151	60.6
	Female	98	39.4
Year of presentation	2011	50	20.1
	2012	57	22.9
	2013	47	18.9
	2014	45	18.1
	2015	50	20.1
*Zoba*/region of origin	Anseba	34	13.7
	Maekel	84	33.7
	North Red Sea	26	10.4
	South Red Sea	17	6.8
	Debub	50	20.1
	Gash Barka	38	15.3

Measurement of distance VA using standard chart was available in 163 (65.5%) children. Out of remaining 86 children, 62 (24.9% of total 249) were believed sighted (Table 2). Multinomial logistic regression showed that children older than 5 years of age were more likely to be in blind category compared to children 5 years or less age (odds ratio (OR): 2.3, *p* = 0.002).

**Table 2:** Visual acuity categories (*N* = 249)

Category of Vision loss (distance VA level)	Number (%)
Visually impaired	125 (50.2)
Vision Impairment (< 6/18- ≥ 6/60)	50 (20.1)
Severe Vision Impairment (< 6/60- ≥ 3/60)	13 (5.2)
Believed sighted (child fixates and follows light or object)	62 (24.9)
Blind	124 (49.8)
Blindness (< 3/60-Light Perception)	84 (33.7)
Blindness (No Light Perception)	16 (6.4)
Believed Blind (child does not fixate and follow light or object)	24 (9.6)

The anatomical sites of abnormalities causing vision loss are presented in Table 3. Lens was the leading site (66 children, 26.5%) with cataract (15 pseudophakia, 11 post-cataract surgery aphakia and 34 un-operated) as the major cause (60 children, 24.1%). Cornea was the second leading site of vision loss present in over 22% of all the cases. This included mainly the corneal scars present since birth (19, 7.6%) and irregular cornea resulting from keratoconus (4.8%). The other leading conditions responsible for vision loss were refractive error/amblyopia (12.1%), phthisis bulbi (6.4%), buphthalmos/glaucoma (5.2%), nystagmus (4.8%) and cortical blindness following meningitis and other systemic ailment (3.6%). Among the children with retinal disorders (8.4%), retinitis pigmentosa, retinal dystrophies, retinopathy of prematurity (ROP) and retinal detachment were noted in almost equal number of children (about 2%). Similarly, optic nerve lesions were responsible for vision loss in 7.2% of children, majority of which were present since birth. Intracranial space-occupying lesions, metabolic disorders, suspected toxicity of systemic drugs during gestational period were the causes of optic atrophy in the remaining cases. Multinomial logistic regression analysis showed that females had more number of retinal conditions than males (OR: 4.5, *p* = 0.01) and children from Maekel *Zoba* had higher number of normal globe conditions than other regions (OR: 2.9, *p* = 0.03). While corneal conditions (OR: 2.8, *p* = 0.03) and lens abnormalities (OR: 2.7, *p* = 0.05) were significantly more in children older than 5 years of age than younger children, reverse was observed for optic nerve conditions (OR: 21.5, *p* = 0.005).

**Table 3:** Anatomical site for vision loss (*N* = 249)

Anatomical Categories	Specific conditions	Frequency	%	Cumulative number (%)
Whole Globe	Phthisis	16	6.4	34 (13.7)
	Anophthalmos/ Microphthalmos	5	2.0	
	Buphthalmos/Glaucoma	13	5.2	
Cornea	Scar	39	15.7	55 (22.1)
	Keratoconus	12	4.8	
	Staphyloma	4	1.6	
Lens	Cataract (including 15 operated cataracts)	49	19.7	66 (26.5)
	Aphakia (including 11 post-cataract surgery aphakia)	14	5.6	
	Dislocated lens	3	1.2	
Uvea	Aniridia	2	0.8	4 (1.6)
	Uveitis	2	0.8	
Retina	Retinitis Pigmentosa	6	2.4	21 (8.4)
	Macular dystrophy	4	1.6	
	Retinopathy of Prematurity (ROP)	5	2.0	
	Retinal Detachment	4	1.6	
	Retinoblastoma	2	0.8	
Optic Nerve	Atrophy	16	6.4	18 (7.2)
	Aplasia/Hypoplasia	2	0.8	
Normal Globe	Refractive error/Amblyopia	30	12.1	51 (20.5)
	Cortical vision loss	9	3.6	
	Nystagmus	12	4.8	
Total		249	100.0	249 (100)

The etiological factors responsible for vision loss could not be classified in 137 (55.0%) children. This category mainly included congenital cataract (22.5%), corneal scars (7.6%) and glaucoma (5.2%), where exact underlying etiology could not be determined based on the information available on the MRCs. In remaining cases, where a cause of reduced vision was attributed, childhood factors were the main etiology (34.5%) (Table 4). Majority of childhood conditions were refractive error/amblyopia (12.1%), trauma (10.4%) and keratoconus (5.6%). The tissue damage mentioned in the MRCs of traumatic cases included corneal scar (2.8%), phthisis bulbi (4.4%), uveitis (0.8%), lens disorder (2.4%) and retinal detachments (0.8%). In 26 MRC, where trauma was the etiological factor, blast injury was mentioned in 12 (4.8% of 249) MRCs. Children from peripheral regions were more likely to have hereditary (OR: 15.8, *p* = 0.03) and normal globe conditions (OR: 4.1; 0.03) compared to children from central Maekel region. Similarly, hereditary conditions (OR: 90.0, *p* = 0.002); childhood factors (OR: 29.1, *p* = 0.02) and unknown etiological factors (OR: 12.5, *p* = 0.01) were more likely to be present in older children (> 5 years of age) than in younger children.

**Table 4:** Etiology for vision loss for the child (*N* = 249)

Etiological Classification	Specific conditions	Frequency	%	Cumulative number (%)
Hereditary	Suspected autosomal dominant (cataract and dislocated lens)	4	1.6	10 (4.0)
	Unspecific: Retinitis pigmentosa	6	2.4	
Intrauterine	Suspected drug toxicity	2	0.8	5 (2.0)
	Rubella retinopathy	1	0.4	
	Others	2	0.8	
Perinatal/Neonatal	Ocular infections including Ophthalmia neonatorum	6	2.4	11 (4.4)
	Retinopathy of Prematurity (ROP)	5	2.0	
Childhood	Vitamin A Deficiency (VAD)	5	2.0	86 (34.5)
	Keratoconus	12	4.8	
	Harmful traditional eye medication practice	2	0.8	
	Measles: corneal scar	1	0.4	
	Meningitis	3	1.2	
	Trauma (cataract, corneal scar and phthisis bulbi, retinal detachment, uveitis)	26	10.4	
	Refractive error/Amblyopia	30	12.1	
	Others	7	2.8	
Unknown	Cataract (11 post cataract surgery aphakia, 30 un-operated congenital cataract and 15 operated congenital cataract)	56	22.5	137 (55.0)
	Glaucoma	13	5.2	
	Corneal Scar	19	7.6	
	Cortical blindness	6	2.4	
	Optic atrophy	9	3.6	
	Congenital globe deformities	8	3.2	
	Nystagmus	12	4.8	
	Others	14	5.6	
Total		249	100.0	249 (100.0)

Altogether, 172 (69.1%) children had potentially avoidable vision loss; 20.5% preventable and 48.6% treatable (Table 5). The preventable conditions included trauma (including traumatic lens disorders) (10.4%), perinatal ocular infections (2.4%), VAD (2.0%), ROP (2.0%), harmful traditional eye medication practice (0.8%), rubella (0.4%) and measles (0.4%). Five cases of vision loss (2.0%) following meningitis or systemic ailment were also considered potentially preventable. Lenticular disorder (24.1%) and refractive error (12.0%) were the major treatable conditions. Other potentially treatable conditions included glaucoma (5.2%), keratoconus (4.8%), retinal detachment (1.6%) and uveitis (0.8%). While avoidable blindness was observed more in children older than 5 years of age (OR: 2.4, *p* = 0.002), no other significant differences in avoidable vision loss were found in between the demographic groups.

**Table 5:** Avoidable nature of vision loss (*N* = 249)

Nature of vision loss	Specific conditions	Frequency	%	Cumulative Number (%)
Potentially preventable	Trauma (including traumatic lens disorder)	26	10.4	51 (20.5)
	Perinatal ocular infections (including Ophthalmia Neonatorum)	6	2.4	
	Vitamin A Deficiency (VAD)	5	2.0	
	Retinopathy of Prematurity (ROP)	5	2.0	
	Cortical blindness following systemic ailment	2	0.8	
	Post meningitis optic atrophy	3	1.2	
	Harmful traditional eye medication practice	2	0.8	
	Rubella	1	0.4	
	Measles	1	0.4	
Potentially treatable	Lens disorders (excluding traumatic lens disorders)	62	24.9	123 (49.4)
	Glaucoma	13	5.2	
	Refractive error/amblyopia	30	12.0	
	Keratoconus	12	4.8	
	Retinal detachment	4	1.6	
	Uveitis	2	0.8	
Unavoidable		75	30.1	75 (30.1)
Total		249	100.0	249 (100.0)

## Discussion

This study identified the causes of vision loss in children attending the national referral eye hospital, with country’s only pediatric ophthalmology services. This method allowed us to examine these causes in a short period of time and with minimal human and financial resources. However, this methodology has certain limitations including incomplete records, selection and information bias and poor standardization because of multiple examiners. Information mentioned in the records was limited, which narrowed the conclusion derived from the retrospective data. It was not possible for us to compare the demographic details of the visually impaired children with that of all the children (22,509) who attended the hospital because of lack of proper recording system at the hospital. In addition, some of the other studies compared here used different methodology such as blind school or population survey and only report details of severe vision impairment and blindness which may not allow a direct comparison with our findings. Despite these limitations, this study provides valuable information on the causes of childhood impairment in Eritrea. Along with the information obtained the children in the country’s only school for the blind [10, 11], the findings of this study can be used as a strong evidence to formulate the programs to address such causes in this community.

Higher number of boys in our study is likely to be associated with poor access of females of all age groups to health care services. It has been observed in some African countries that more boys visit hospitals for cataract surgery than girls even though the prevalence of cataract blindness in both genders is similar [14] and parents tend to give more priority to boys than girls [15]. In addition to gender differences, age at first presentation was significantly high i.e. mean age at presentation was over 7 years and over 40% of the children were of >10 years of age. Late age of presentation in our study may be because of lack of preschool vision screening program, parental unawareness, centralized location of the facility and the fact that many of the causes identified are associated with late onset of vision loss such as keratoconus, retinitis pigmentosa and pathological myopia. It should also be noted that BEH is the only eye hospital with pediatric specialty receiving referral from all over the country. A long travel required to reach this center imposes significant financial and other challenges to the visually impaired children and their families. Thus development of secondary centers and training in pediatric eye care for different cadres of eye care at regional level need be considered.

Over one quarter of vision loss was due to lenticular disorders. Much lower proportion of lens related vision loss has been reported from neighboring African and other countries [4, 7, 8, 16–19]. Many of these cataract cases were un-operated for the reasons of associated complications and poor visual prognosis. For the operated cases, delayed presentation, surgical complications and poor rehabilitation after surgery might be the reasons for vision impairment even after cataract extraction. Further strengthening of existing facilities; development of new surgical centers and an effective mechanism to identify and refer children with cataract in the community could address this issue.

Corneal disorders (mainly opacities) are major concerns in developing countries and are responsible for up to two-thirds of childhood vision loss whereas only about 5% of vision loss in children is attributed to these causes in developed countries [4, 7, 17, 19]. The survey of the blind school children in Eritrea has also demonstrated low proportion of corneal blindness, especially that related to VAD and measles [10]. Majority of the cornea related vision loss in our study was caused by corneal scars which were present since birth, and causes such as VAD, measles, harmful traditional medications and infection were relatively fewer. The other cause of corneal vision loss was the irregular cornea resulting from keratoconus. This condition has been shown to cause vision loss as high as 11% in hospital based study from Yemen [8]. Many of these cases could have been managed to a good level of distance visual acuity using appropriate contact lenses. Contact lens services are almost non-existent in the country mainly due to lack of trained human resources, which is expected to be addressed by current optometry training program.

Phthisis bulbi (congenital, post-infection or traumatic) was the other major condition causing vision loss (6.4%), which compares to 11.3% in the blind school survey [10], Similarly, the proportion of other globe deformities (anophthalmos and microphthalmos) was significantly lower in this study (2%) compared with the blind school survey (14.1%) [10]. These conditions might have been underrepresented as many parents might consider a shrunken eye as already damaged and do not feel a need to visit a hospital for further possibilities of management.

Retinal disorders were responsible for vision loss in comparatively fewer children in this study (just the half of compared to the blind school survey [10]). Proportion of retinal blindness varies in between different countries and ranges from just over 2% in poor countries [19, 20] to almost 50% in high income countries [4]. While retinitis pigmentosa and retinal dystrophies are common retinal causes of vision loss in children [21], ROP is an emerging condition especially in children in middle-income countries like Turkey [7] and Surinam [11]. With improving neonatal care for preterm babies in Eritrea [22], a rise in the number of children with ROP can be expected. The national blindness control program and BEH need to collaborate to start a stringent screening program, treatment facilities and training of ophthalmologists and pediatricians. Interestingly, no case of vision loss attributed to retinal toxoplasma scar was identified in this study in contrast to the findings of the blind school survey, where this condition was responsible for 8.5% of severe vision impairment and blindness [10]. We are unable to provide any robust reason for the difference of retinal toxoplasma scar related vision loss observed in two different samples from the same country.

Sequel of childhood ocular injury was a leading etiological factor responsible for vision loss in children attending BEH. The blind school survey had also identified trauma (especially the blast injuries) as one of the leading etiologies of vision loss in children [10]. Ocular injuries have been shown to cause generally 1 to 5% of all children in attending blind schools [9, 16, 19, 23, 24], however, this proportion is drastically high (up to 32%) in countries affected by civil wars [25, 26]. As half of the ocular injuries were the result of land mine explosion, there is an urgent need of creating awareness and removing mines in civilian areas to prevent such accidents.

On the other hand, the proportion of blindness due to VAD, measles and perinatal infections is lower than that for countries with poor socioeconomic status [4, 19]. The lower proportion of such etiologies in children less than 5 years of age is an encouraging situation indicating the effects of successful vitamin A supplementation, measles immunization and improved perinatal care services in Eritrea [22]. However, the presence of these conditions also suggests that Eritrean blindness control program still needs to work on these preventable causes of corneal scars. Vision loss due to harmful traditional medication was present in only two children which is believed to be underrepresented because of missing case history in some of the cases of corneal scars of unknown etiology. As this condition has been shown to be responsible for up to 6% of vision loss in children [16], programs to increase awareness against the practice of harmful traditional eye medication with the involvement of traditional healer have been suggested [27].

Hereditary and intrauterine factors were identified in relatively few MRCs. Details of past medical history, family history and other investigations are required to ascertain such factors leading to vision loss. As many MRCs lacked such details, these cases were labeled as ‘unknown etiology’. This observation is consistent with results from other studies and indicates the need of detailed history and advanced examinations in order to identify the exact etiology of vision loss in these children. As identification of the etiologies of vision loss helps in developing targeted blindness control programs, presence of large number of cases with unspecific underlying etiology in both blind school survey and this study may adversely impact the development and implementation of such programs [4, 10]. Proper record keeping of the findings in children with vision loss based on WHO classification system [13] was recommended to the hospital as it will allow authorities a periodic evaluation of changing pattern of childhood blindness in the country.

Avoidable vision loss was present in about 70% of the children which is comparable with the reports from developing countries [7, 19, 24]. The higher number of avoidable conditions in this study compared to blind school survey is due to the presence of large number of vision impairment caused by refractive errors (including those caused by keratoconus) which was not found in the blind school [10]. An effective vision screening program needs to be implemented along with the provision of glasses for those having a significant refractive error. However, as the refractive care services are often centralized and the optical dispensing services are limited in the country [28], authorities need to further strengthen the vision centers as well as develop human resources to provide proper refractive care and optical dispensing services to children. At the same time, tertiary level services including contact lens, vision therapy and vision rehabilitation need to be developed which are lacking in this country [29]. For cataract and glaucoma in children, the management is usually challenging and visual prognosis depends on several factors. To address vision loss due to these conditions, early identification and referral mechanism at primary level and specialty ophthalmology services including medical, surgical and rehabilitative facilities need to be effectively developed and implemented [4].

On the other hand, damage to the cornea and the whole globe due to trauma was the leading preventable cause of vision loss followed by perinatal ocular infection, VAD and ROP. For the control of vision loss due to each of these conditions, there is need of specific programs at different levels including awareness creation, clearing the mine fields, nutritional supplement and immunization. Further development of pediatric health facilities could help reduce the number of vision loss due to complications of systemic illness such as meningitis.

## Conclusion

Our hospital based data on causes of vison loss in children identified that nearly two thirds of the vision loss is avoidable in nature. While congenital anomalies such as cataract, corneal scars, glaucoma remain the leading cause of vision loss, large proportion of vision loss is also due to the sequels of ocular trauma and refractive errors. VAD, measles and infections, although relatively few, remain important causes of vision loss in these children. To address these avoidable conditions, preventive measures (trauma prevention, nutrition, and awareness) and curative services (surgery, refraction and optical dispensing) need to be developed. At the same time, for the conditions where effective treatment is not available, rehabilitative services can be useful.

## Abbreviations

BEH: Berhan Aiyni National Referral Hospital
MRC: Medical record card
ROP: Retinopathy of prematurity
VA: Visual acuity; VAD: Vitamin A deficiency
